# Radiation-Induced Wavelength Shifts in Fiber Bragg Gratings Exposed to Gamma Rays and Neutrons in a Nuclear Reactor

**DOI:** 10.3390/s25020323

**Published:** 2025-01-08

**Authors:** G. Berkovic, S. Zilberman, Y. London, M. Rosenfeld, E. Shafir, O. Ozeri, K. Ben-Meir, A. Krakovich, T. Makmal

**Affiliations:** Soreq NRC, Yavne 81800, Israel

**Keywords:** fiber Bragg gratings, ionizing radiation, gamma rays, neutrons

## Abstract

Fiber Bragg gratings (FBGs) inscribed by UV light and different femtosecond laser techniques (phase mask, point-by-point, and plane-by-plane) were exposed—in several irradiation cycles—to accumulated high doses of gamma rays (up to 124 MGy) and neutron fluence (8.7 × 10^18^/cm^2^) in a research-grade nuclear reactor. The FBG peak wavelengths were measured continuously in order to monitor radiation-induced shifts. Gratings inscribed on pure silica core fibers using near-IR femtosecond pulses through a phase mask showed the smallest shifts (<30 pm), indicating that these FBGs are suitable for temperature measurement even under extreme ionizing radiation. In contrast, the pointwise inscribed femtosecond gratings and a UV-inscribed grating showed maximal shifts of around 100 pm and 400 pm, respectively. Radiation-induced red shifts are believed to arise from gamma radiation damage, which may partially recover after irradiation is stopped. At the highest neutron exposures, grating peak blue shifts started to appear, apparently due to fiber compaction.

## 1. Introduction

Fiber Bragg gratings (FBGs) are popular sensors exhibiting well-characterized responses (wavelength shifts) to various physical parameters such as temperature and strain [1,2,3]. Furthermore, these sensors have additional attractive features including their multiplexing ability, immunity to electro-magnetic interference, and ability to be deployed in remote and hostile surroundings. Thus, FBGs appear to be attractive candidates for the sensing and monitoring of temperature and structural integrity in nuclear reactors and other ionizing radiation-rich environments [2,3].

We recall that FBG reflectivity peaks at the wavelength equal to 2 nΛ, where n is the effective fiber refractive index, and Λ is the grating periodicity. If either (or both) of these quantities change with temperature, strain, humidity, etc., the FBG peak changes, enabling the monitoring of these environmental factors. For example, temperature monitoring exploits the fact that standard C-band FBGs in single-mode silica fiber near room temperature shift by around 10 pm/°C [3]. Using commercially available instrumentation with peak detection methods and a resolution of 1 pm, a nominal temperature resolution of 0.1 °C is achievable.

The fact that the FBG peak wavelength is sensitive to several external physical parameters is both an advantage (widespread applicability) and a disadvantage (cross-sensitivity). In some cases, cross-sensitivity can easily be overcome—e.g., a loosely or freely mounted FBG will be immune to strain, enabling a pure temperature response [4]. However, the cross-sensitivity issue raises the question of whether exposure to ionizing radiation itself can impart a shift in the FBG peak wavelength which, if so, will complicate temperature measurements even with a freely mounted FBG in a radiation-rich environment.

The simplest solution to eliminate the possibility of temperature–ionizing radiation cross-sensitivity in FBGs is to have an FBG which is “unaffected” by ionizing radiation [5]. The term “unaffected” is written in quotation marks to stress that this is a qualitative term only, subject to interpretation. For a given application, one should define an acceptable limit to a radiation-induced FBG peak shift under the appropriate radiation levels. For example, if temperature determination with 2 °C accuracy is required, then an FBG whose peak is shifted by less than 20 pm under the radiation exposure conditions of the application should suffice.

Radiation’s effects on multiple types of optical fibers have been widely studied and characterized and reviewed [6,7]. The main aim in most of these studies was to examine the radiation-induced attenuation (RIA) of the transmission through the fiber. This attenuation is connected to the radiation-induced formation of structural defects in the fiber and can be correlated with dopants present in the fiber core [7]. Fibers with pure silica cores, and fluorine-doped cores, were found to have much higher resistance to ionizing radiation (radiation hard) than fiber with Ge-, P-, and Al- doped cores.

The first reports of ionizing radiation’s effects specifically on FBGs in silica fibers appeared about 20 years ago and studied [8] the gratings in use at that time—namely, FBGs inscribed by the UV irradiation of hydrogen-loaded fibers. Indeed, the first question studied was whether the gratings would be erased by ionizing radiation (they were not), and the fact that the grating peaks exhibited rather large red shifts (hundreds of pm to nm) was secondary.

More recently, FBG inscription via the multi-photon absorption of near-IR femtosecond pulses has become common. Femtosecond gratings should be differentiated by their inscription technique. They may be inscribed either through a phase mask or by focusing pulsed IR light sequentially (stepwise) at different points along the fiber core axis [2]. This point-by-point inscription can be modified to line-by-line and plane-by-plane inscription by the transverse scanning of the focused inscription point or by using a lens with a longer focal range [9]. Pointwise femtosecond (fsec) inscription, as opposed to phase mask inscription, leads to higher peak power intensities and different mechanisms for refractive index modulation, including void formation [10]. This differentiation has led to the power-based grating classification of FBGs as Type I, II, or III FBGs [3,10,11].

**Figure 1:**
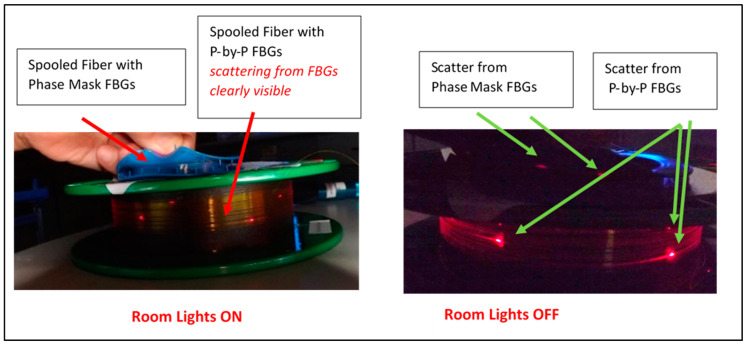
A visual demonstration of the difference between femtosecond phase mask and femtosecond point-by-point FBGs. (**left**) The green spool is a connectorized fiber containing point-by-point FBGs, and the smaller blue spool is a fiber with phase mask FBGs. Both fibers are illuminated using red light from fiber fault locators. In a bright room, scattering is visible from the point-by-point FBGs but not from the phase mask FBGs. (**right**) Weak scattering from the phase mask FBGs can only be observed when the room is completely darkened.

Point-by-point femtosecond FBGs with void formation can often exhibit enhanced scattering, observable visually by side-viewing, when visible light propagates through the fibers [12]. In Figure 1, scattering from fibers with phase mask and point-by-point femtosecond gratings is compared when red light is injected into fibers using a commercial fiber fault locator (a red diode source). The point-by-point FBGs exhibit much stronger scattering, visible to the naked eye even in bright room light. Scattering from the phase mask FBGs can only be seen when the room is in darkness.

A series of studies of irradiation’s effects on femtosecond (fsec) FBGs was performed and reviewed [11] by Morana et al., showing that under ionizing radiation, femtosecond FBGs shift less than UV-inscribed gratings. In addition, differently inscribed fsec FBGs under identical irradiation conditions were compared. It should also be pointed out that the underlying fiber on which the FBG is inscribed is important—UV gratings are necessarily inscribed on photosensitive fiber, while femtosecond gratings may be inscribed on “radiation-resistant” fibers (pure silica cores and F-doped silica cores). Thus, ionizing radiation is likely to modify the underlying fiber refractive index to a greater extent in the former.

Although the above-mentioned review [11] encompasses numerous studies, they are mostly concentrated on X-ray and γ-ray irradiation at dose levels of up to hundreds of kGy. Here, we report and extend similar comparisons for various FBGs irradiated by neutrons and γ-rays in a research-grade nuclear reactor, at dose levels exceeding 100 MGy.

## 2. Experimental Procedure

**Figure 2:**
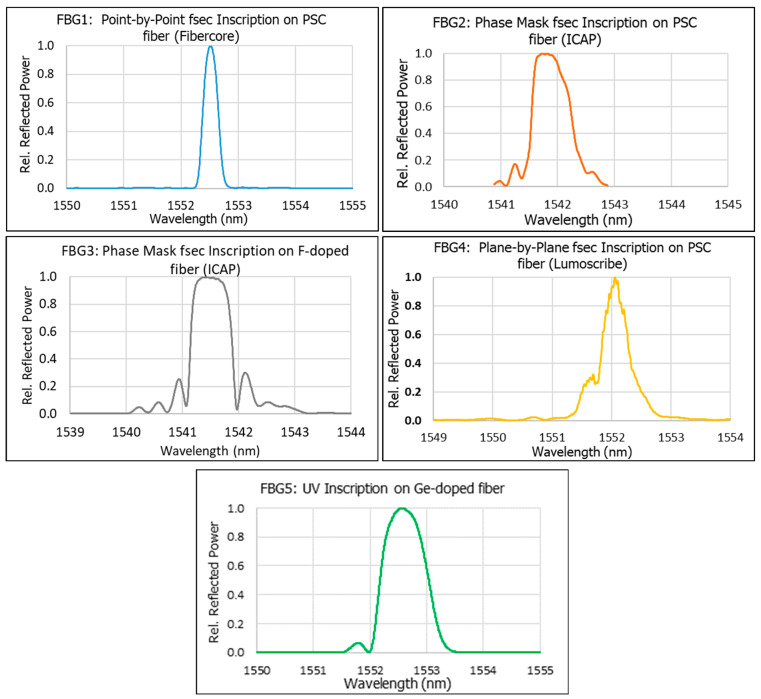
The reflection spectra of the FBGs used in this study, as measured by an Optical Spectrum Analyzer (OSA). All graphs are presented with the FBG reflected power normalized with respect to its maximum. In all graphs, the full scale along the x-axis is the same (5 nm), enabling an easy comparison of the bandwidths of the different FBGs.

Our experiment involved 5 FBGs in the 1550 nm band which were irradiated in a Soreq Nuclear Reactor [13,14]. The spectra of the 5 FBGs—measured using an Optical Spectrum Analyzer (Yokagawa AQ6370C (Musashino, Japan) at 20 pm resolution)—are given in Figure 2. Four of the FBGs were femtosecond near-IR FBGs inscribed on “radiation-resistant” single-mode fibers; two FBGs were inscribed via a phase mask and the other two by stepwise inscription (either point-by-point or plane-by-plane) through the fiber coating. The fifth FBG was inscribed by UV on a Ge-doped fiber with hydrogen loading. The fsec phase mask FBGs were inscribed at the Israeli Center for Advanced Photonics (ICAP), and the other FBGs were obtained commercially from Fibercore (Southampton, UK), Lumoscribe (Paphos, Cyprus), and O/E Land (Montreal, QC, Canada)—see Table 1. Note that FBG1, FBG2, and FBG4 were all inscribed on the same pure silica core single-mode fiber (Fibercore SM1250SC), while FBG3 was inscribed on a F-doped silica core single-mode fiber purchased from iXBlue (Paris, France) [14]. All fibers were polyimide-coated (recoated in the case of the UV grating) with a typical thickness of 10 μm.

**Figure 3:**
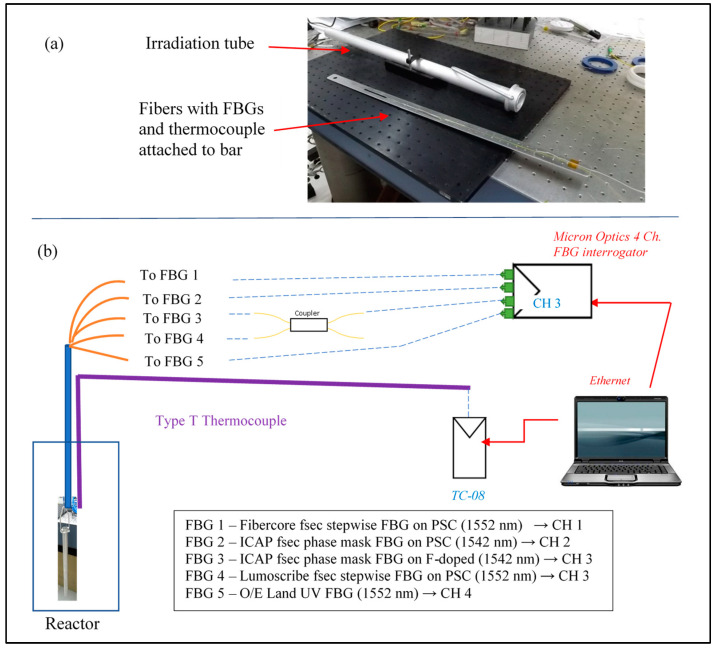
(**a**) A photograph of fibers on an aluminum bar and the irradiation tube into which they are placed and lowered into the reactor. The five FBGs and the thermocouple sensing head are all positioned (to within 1 cm) at the same location on the bar to ensure equal exposures during the experiment. (**b**) Experimental set-up for monitoring FBG peaks and temperature during the experiment.

In earlier publications (describing measurements of radiation-induced attenuation) we have described the Soreq Research Reactor and the method for assembling and inserting fibers into the reactor core, as well as performing on-line monitoring during reactor operation [13,14]. The FBGs, at the end of 12 m fiber pigtails, are attached to an aluminum bar which is inserted into an irradiation tube which can be lowered into the reactor core (see Figure 3a). Note that the reactor core is at the bottom of a pool, and thus, the fibers are in contact with water. Since polyimide coatings can absorb water and strain the FBG in the fiber core [15], it was important to check the magnitude of this effect on the FBGs employed prior to this experiment: saturating the fibers with water caused the FBG peak wavelengths to red-shift by 20–30 pm. In the current experiment, the fibers were connected to a multi-channel FBG interrogator (Micron Optics SM130) to measure the FBG reflection peaks, rather than measuring optical power transmission, as in our earlier publications [13,14]. A schematic of the experimental set-up is given in Figure 3b. In order to monitor the five FBGs in the 4-channel interrogator, two spectrally separated FBGs were monitored on the same channel (channel 3) by adding a standard 2 × 2 single-mode coupler. A type-T thermocouple—verified for stability in high-radiation environments [16]—was also inserted with the fibers to monitor the temperature during this experiment using a TC-08 data logger (pico Technology, Cambridgeshire, UK). The FBG peaks and temperature were logged every 10 min during this experiment using the manufacturer-supplied interrogator and thermocouple software.

As in our previous study [14], the fibers may be placed in two locations in the reactor with different radiation dose levels: the “weaker” irradiation site gives exposures of 0.7 MGy/h of γ-rays and 10^13^ neutrons/cm^2^·s, while the exposures in the “stronger” location are 4 MGy/h of γ-rays and 8 × 10^13^ neutrons/cm^2^·s. The reactor is typically operated in a shift of several (4–10) h and then shut down until the next operation. During the down times, the fibers remained in the core, and monitoring continued.

## 3. Results

In total, the FBGs were exposed to ionizing radiation during eight operating shifts of the reactor, spanning several weeks. In the first four shifts, the fibers were positioned in the “weaker” irradiation site, and in the last four shifts, they were positioned in the stronger site. Note that some results from the early operating shifts were presented in conference proceedings [17].

FBG peaks and temperature were monitored both during reactor operation shifts and during the shutdowns between operations. Note that the FBG interrogator used (Micron Optics SM130) gives the FBG peaks as its output, rather than the full spectra shown in Figure 2. The FBG peak readings are converted to shifts relative to their reference values. The FBG reference values are the peak maxima measured immediately prior to the first reactor operation, at which time the fibers had been in the (shutdown) core for over 24 h, allowing for the equilibration of the polyimide coatings with the surrounding water, whose temperature was 26.4°. Since the temperature varied during the experiment (by up to 5°), subsequent FBG readings were corrected on the basis of the thermocouple reading with a correction factor of 10 pm/° to the reference temperature of 26.4°. Considering all the experimental uncertainties—thermocouple (0.2° = 2 pm), the interrogator (<5 pm), and the temperature correction factor of 10 pm/° having an uncertainty of ±0.5 pm/° thus contributing another 2 pm uncertainty for a correction of at most 5°—the total uncertainty in wavelength shift should be around 5 pm.

**Figure 4:**
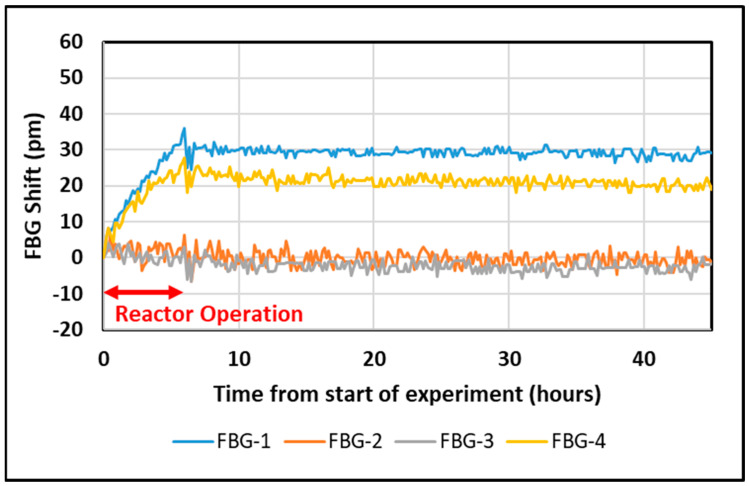
Changes in the FBG peak wavelengths (corrected for temperature variations) during and after the first reactor operation shift.

Data from during and after the first reactor operation shift are shown in Figure 4. The reactor operated for 6 h, and with the FBGs in the “weaker” irradiated site, the total exposure was 4.2 MGy of γ radiation and 2.2 × 10^17^ neutrons/cm^2^.

As can be seen in Figure 4, the two phase mask fsec gratings (FBG 2 and FBG 3) showed no discernable shifts relative to the approx. 5 pm measurement noise (in agreement with the above estimate), while FBG 1 and FBG 4, which are two stepwise fsec gratings, showed shifts of tens of pm to longer wavelengths (red shift) occurring in the clearly defined time windows of the reactor’s operation. After the reactor was shut down, little or no change was observed. The UV-inscribed grating FBG 5 is not shown for clarity, but its shift was of the order of 100 pm. The larger shift in the UV-inscribed grating is a manifestation of the difference between gratings inscribed on Ge-doped photosensitive fibers versus radiation-resistant fibers and is consistent with earlier publications [8,11].

**Figure 5:**
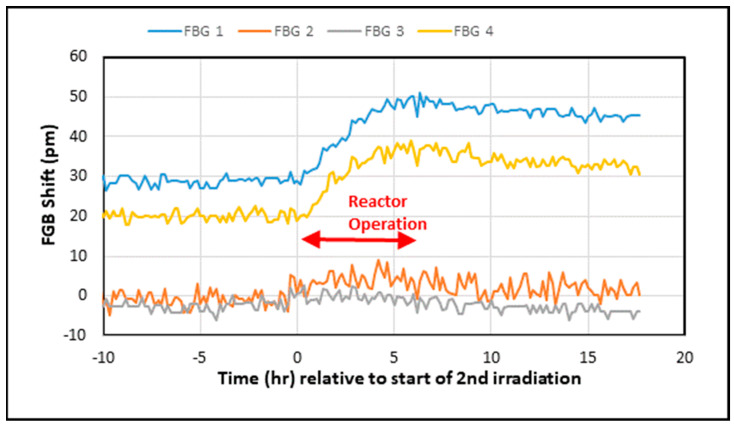
Accumulated changes in the FBG peak wavelengths (corrected for temperature variations) before, during, and after the second reactor operation shift.

Several days later, the fibers were exposed to further radiation during the next reactor operation. As can be seen in Figure 5, during this operation, the peak wavelengths of FBG 1 and FBG 4 continued to grow until the reactor operation ceased. As in the first operation, FBG 2 and FBG 3 again did not exhibit any measurable shift (relative to the experimental noise level).

The FBGs were exposed during two further reactor shifts in the “weaker” irradiation site (four shifts in total). The spectral shifts in the two phase mask fsec gratings (FBG 2 and FBG 3) were still negligible compared to the 5 pm uncertainty, while the shifts in the other FBGs increased monotonically to about 70 pm (FBG 1), 50 pm (FBG 4), and 220 pm (FBG 5).

**Figure 6:**
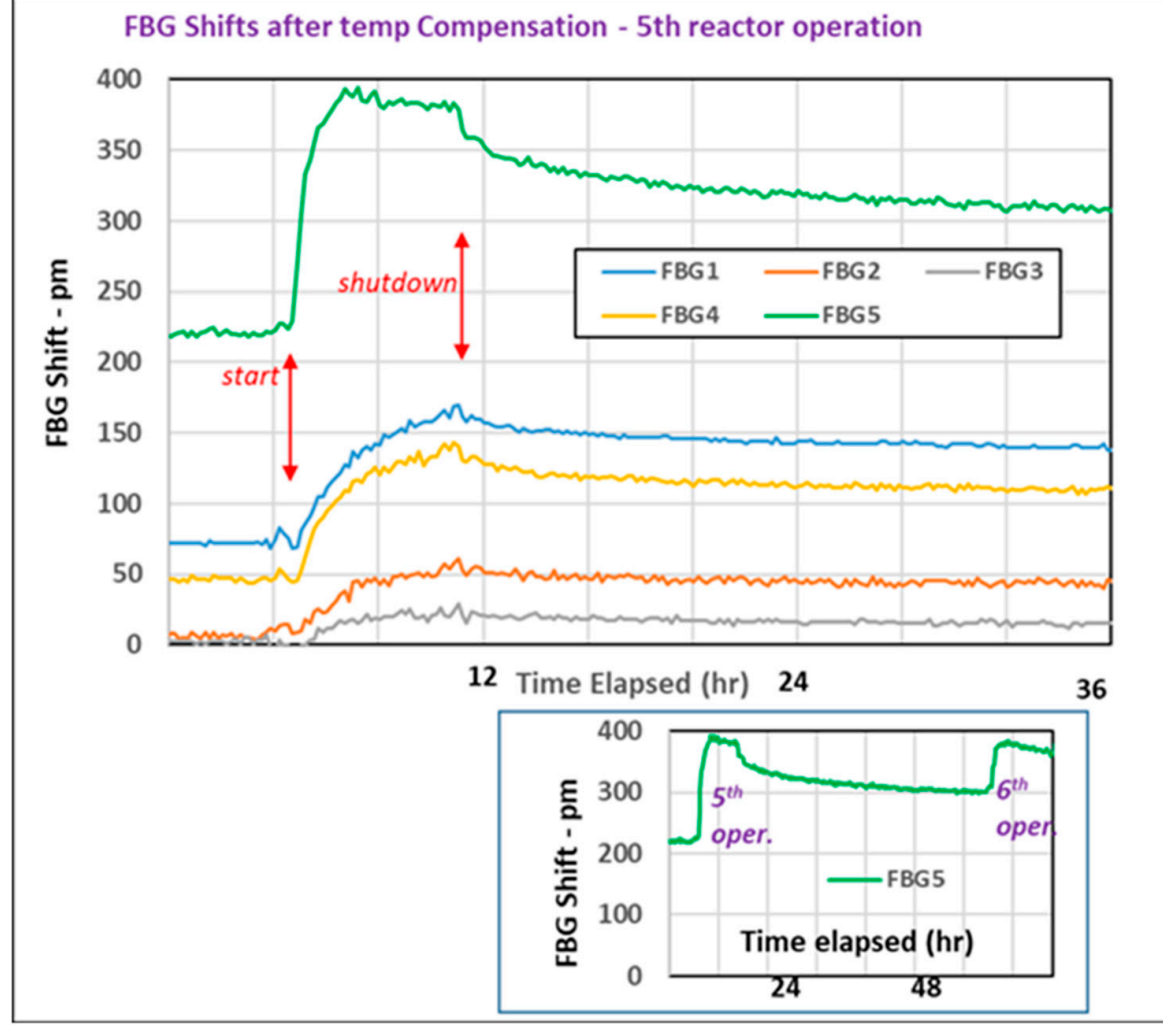
Changes in the FBG peak wavelengths (corrected for temperature variations) during and after the fifth reactor operation shift (first operation in the stronger irradiation site). The inset at the bottom also shows the partial recovery of the peak shift after shutdown and rapid erasure at the start of the next shift.

The next (5th) and subsequent (6th–8th) irradiations were performed in the stronger irradiation site (4 MGy/h of γ-rays and 8 × 10^13^ neutrons/cm^2^·s). During the fifth irradiation (Figure 6), further red shifts were observed, including smaller shifts for the two phase mask fsec gratings (FBG 2 and FBG 3) which had been highly stable until then. After reactor shutdown, the FBG wavelength shifts “recovered” to some degree—i.e., their red shifts decreased. However, upon the next irradiation shift 2 days later, the “recovery” was rapidly erased—shown for FBG 5 at the bottom of the figure. This erasure of recovery upon the reactor restart is analogous to what was observed in radiation-induced attenuation (RIA) studies of fiber transmission [13,14]. This recovery/erasure is attributed to the annealing of radiation-induced defects to metastable species which are rapidly restored to defects when the ionizing radiation is resumed. We can assume that the process will also lead to metastable refractive index changes in the underlying fiber, causing the FBG peak to recover slowly during annealing and being erased when exposure resumes.

**Figure 7:**
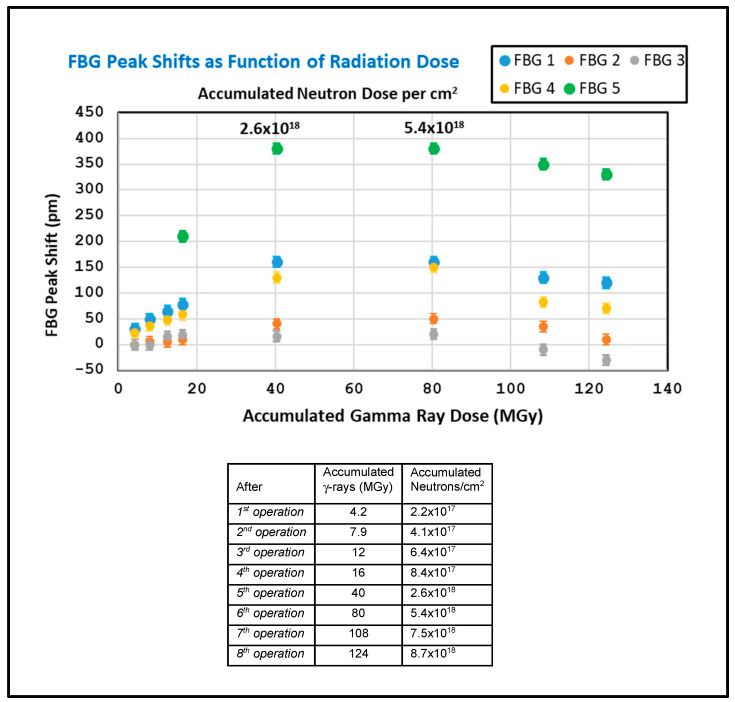
Cumulative shifts in FBG peak wavelengths at the conclusions of all reactor operations expressed as a function of radiation exposure. The bottom horizontal axis is the accumulated dose of γ-rays, and the neutron doses accumulated in parallel are given on the top axis and in the table at bottom.

Figure 7 displays data showing the FBG peak shifts measured immediately after each shutdown at the end of the eight irradiations, plotted against the total radiation exposure. As can be seen, monotonic red shifts are observed for the first five exposures, followed by “saturation” in the sixth exposure and blue shifts during the final two exposures. The total radiation exposure was 124 MGy of γ radiation and 8.7 × 10^18^ neutrons/cm^2^. In order to confirm that the blue shifts are real, it was necessary to verify that the T-type thermocouple used for temperature compensation was still functioning correctly after the high irradiation exposure (much higher than that in the reported qualification [16]). The confirmation was performed after the final irradiation cycle when the irradiated fibers and samples were raised out of the reactor core into the topmost part of the reactor pool where the thermocouple gave correct temperature readings, agreeing with measurements by unexposed thermocouples. The blue shifts observed at the higher doses are consistent with the neutron-induced compaction of the fiber gratings which requires >10^18^ neutrons/cm^2^ [18], while the previously dominant red shifting is associated with defect formation [11,19].

The saturation of the FBG peak red shifts during the sixth exposure and the subsequent blue shifts upon further irradiation may be explained by “competition” between γ radiation damage and neutron compactions. The former leads to red shifting at lower irradiation doses, while the latter causes blue shifting, which becomes dominant at higher doses. All five tested FBGs showed turnover at approximately the same dose, although it appears that turnover occurs in FBG5 slightly before the others. This grating is inscribed in a Ge-doped fiber for which the γ radiation shifts are larger and occur faster.

## 4. Summary and Conclusions

In conclusion, this study demonstrated that the most stable FBGs under our strong ionizing radiation conditions (both γ-rays and neutrons) are those inscribed on radiation hard fibers by the femtosecond phase mask technique. Over the entire regime tested (up to 124 MGy of γ radiation and 8.7 × 10^18^ neutrons/cm^2^), the FBG peak showed radiation-induced shifts of 30 pm or less, meaning that the uncorrected peak shift will measure temperature variation with an accuracy of 2–3 degrees.

Stepwise femtosecond FBGs and UV-inscribed FBGs (which necessarily require photosensitive fibers) show discernable peak shifts both due to refractive index changes from γ-radiation damage and compaction at the highest neutron doses. The former is the strongest for the UV grating on photosensitive fibers versus radiation-resistant fibers for femtosecond FBGs and is somewhat reversible after shutting down irradiation.

The higher resistance to the radiation effects of phase mask-inscribed femtosecond FBGs, relative to femtosecond FBGs inscribed by stepwise exposure to focused pulses, may be explained by the different physical modifications of the fiber material being induced under more extreme laser intensities in the latter. Under focused inscription, material ablation and void formation are expected [3,10,11], and these are qualitatively observed by increased scattering from the FBGs. We should point out that since our sample size is small (two phase mask and two stepwise femtosecond FBGs), it would be helpful if these studies could be expanded to test more gratings from other manufacturers.

To conclude, phase mask femtosecond FBGs on radiation hard fibers should be applicable for use in high-radiation environments as reliable sensors for strain and temperature, due to their very low cross-sensitivity to ionizing radiation.

## Figures and Tables

**Table 1:** FBGs used in this study.

	Supplier	Fiber Type (All Single Mode)	Inscription Technique
FBG 1	Fibercore	Pure Silica Core	fsec; stepwise—point-by-point
FBG 2	ICAP	Pure Silica Core	fsec; phase mask
FBG 3	ICAP	F-doped Silica Core	fsec; phase mask
FBG 4	Lumoscribe	Pure Silica Core	fsec; stepwise—plane-by-plane
FBG 5	O/E Land	Hydrogen-loaded Ge-doped	UV

## Data Availability

The data presented in this study are available on request from the corresponding author.
